# PD-L1 expression in papillary renal cell carcinoma

**DOI:** 10.1186/s12894-016-0195-x

**Published:** 2017-01-13

**Authors:** Takanobu Motoshima, Yoshihiro Komohara, Chaoya Ma, Arni Kusuma Dewi, Hirotsugu Noguchi, Sohsuke Yamada, Toshiyuki Nakayama, Shohei Kitada, Yoshiaki Kawano, Wataru Takahashi, Masaaki Sugimoto, Motohiro Takeya, Naohiro Fujimoto, Yoshinao Oda, Masatoshi Eto

**Affiliations:** 1 Department of Cell Pathology, Graduate School of Medical Sciences, Kumamoto University, Kumamoto, 860-8556 Japan; 2 Department of Urology, Graduate School of Medical Sciences, Kumamoto University, Kumamoto, Japan; 3 Department of Anatomy Histology, Faculty of Medicine Airlangga University, Surabaya, Indonesia; 4 Department of Pathology and Cell Biology, School of Medicine, University of Occupational and Environmental Health, Kitakyushu, Japan; 5 Department of Urology, School of Medicine, University of Occupational and Environmental Health, Kitakyushu, Japan; 6 Department of Anatomic Pathology, Graduate School of Medical Sciences, Kyushu University, Fukuoka, Japan; 7 Department of Urology, Graduate School of Medical Sciences, Kyushu University, Fukuoka, Japan

## Abstract

**Background:**

The immune escape or tolerance of cancer cells is considered to be closely involved in cancer progression. Programmed death-1 (PD-1) is an inhibitory receptor expressed on activating T cells, and several types of cancer cells were found to express PD-1 ligand 1 (PD-L1) and ligand 2 (PD-L2).

**Methods:**

In the present study, we investigated PD-L1/2 expression in papillary renal cell carcinoma (pRCC).

**Result:**

We found PD-L1 expression in 29 of 102 cases, but no PD-L2 expression was seen. PD-L1 expression was not significantly correlated with any clinicopathological factor, including progression-free survival and overall survival. The frequency of PD-L1-positive cases was higher in type 2 (36%) than in type 1 (22%) pRCC; however, there was no significant difference in the percentages of score 0 cases (*p* value = 0.084 in Chi-square test). The frequency of high PD-L1 expression cases was higher in type 2 (23%) than in type 1 (11%), and the frequency of high PD-L1 expression cases was higher in grade 3/4 (21%) than in grade 1/2 (13%). However, no significant association was found between PD-L1 expression and all clinicopathological factors in pRCC.

**Conclusion:**

High expression of PD-L1 in cancer cells was potentially associated to highly histological grade of malignancy in pRCC. The evaluation of the PD-L1 protein might still be useful for predicting the efficacy of anti-cancer immunotherapy using immuno-checkpoint inhibitors, however, not be useful for predicting the clinical prognosis.

**Electronic supplementary material:**

The online version of this article (doi:10.1186/s12894-016-0195-x) contains supplementary material, which is available to authorized users.

## Background

Renal cell carcinoma (RCC) is a common cancer of the kidney, and the three most frequent histological subtypes are clear cell RCC (ccRCC, 70 to 80%), papillary RCC (pRCCc, 10 to 20%), and chromophobe RCC (5%) [1]. Although patients with sporadic RCC of any histological subtype usually show a good clinical outcome, patients with metastatic pRCC show a significantly worse clinical course than patients with ccRCC or chromophobe RCC [2–4]. Recent findings have indicated that *MET* gene　activation, which is known to promote proliferative activity and cell survival, is frequently observed in pRCC, and MET inhibitors have become a new type of therapeutic agent for patients with advanced pRCC [5, 6].

Anti-cancer immune responses were considered to play important roles in preventing cancer progression in RCC [7]; however, immunotherapy against advanced RCC such as interferon therapy and vaccine therapy has shown limited anti-cancer effects over the past fewdecades [8]. In recent years, immuno-checkpoint inhibitors have attracted much attention. Anti-CTLA-4 antibodies, which are used to treat advanced melanoma patients, have been reported to have an excellent therapeutic effect [9]. Subsequently, anti-PD-1 antibody was discovered and used to treat kidney cancer, non-small cell lung cancer and malignant melanoma patients, and superior therapeutic effects have been reported [10]. Some clinical trials demonstrated that combination therapy using anti-CTLA-4 antibody and anti-PD-1 antibody produced significant anti-cancer effects [11]. However, although the antibodies produced excellent therapeutic effects in most patients, no therapeutic effect was observed in some patients. PD-1 ligand 1 (PD-L1) expression in cancer tissues is considered to be a biomarker for predicting the therapeutic effect of immuno-checkpoint inhibitors [12]. Although some studies have demonstrated that a high expression of PD-L1 was associated with poor clinical outcomes [13–17], few studies have investigated PD-L1 expression in pRCC. Therefore, we analyzed the correlation between PD-L1 expression and clinicopathological factors in pRCC.

## Methods

### Patients and Samples

We reviewed 102 cases of pRCC that were excised at Kumamoto University, the University of Occupational and Environmental Health, and Kyushu University between 2001 and 2014. All samples were obtained with informed consent from patients in accordance with the study protocols that were approved by the review board of each university (Kumamoto University Hospital Review Board, Kyushu University Review Board, Review Board of University of Occupational and Environmental Health). Tissue samples of primary site were fixed in 10% neutral buffered formalin and were embedded in paraffin as per a routine method. Nuclear grade and T classification were assessed according to the World Health Organization classification. Patient characteristics, such as age, gender, Fuhrman grade, pathological TNM stage and follow-up data were retrospectively collected.

### Immunohistochemistry

Rabbit monoclonal antibodies against PD-L1 (clone E1L3N) and PD-L2 (clone D7U8C) were purchased from Cell Signaling Technology (Danvers, MA, USA). Briefly, after samples were reacted with primary antibodies, they were incubated with horseradish peroxidase (HRP)-labeled goat anti-rabbit secondary antibodies (Nichirei, Tokyo, Japan). Can Get Signal Solution (TOYOBO, Tokyo, Japan) was used to dilute the antibodies for enhancing the immunoreaction. Reactions were visualized using the diaminobenzidine substrate system (Nichirei). Two pathologists (TM and YK), who were blinded to information about the samples, evaluated the immunostaining of PD-L1/2.

### Cell lines

Two lymphoma cell lines (PD-L1/2-positive cell line; ATL-T, PD-L1/2-negative cell line; DAUDI) were obtained from RIKEN Cell Bank (Tsukuba, Japan), and cell block specimens were used for confirmation of specificities of anti-PD-L1/2 antibodies. Cells were fixed in 10% neutral buffered formalin and cell block samples were embedded in paraffin.

### Statistics

Statistical analysis was carried out with StatMate III (Atoms, Tokyo, Japan). The simultaneous relationships between multiple prognostic factors for survival were assessed using the Cox proportional hazards model with a stepwise backwards reduction. A value of *p* < 0.05 was considered to be statistically significant.

## Results

### PD-L1/B7-H1 expression in pRCC and correlations with clinicopathological factors

**Fig. 1:**
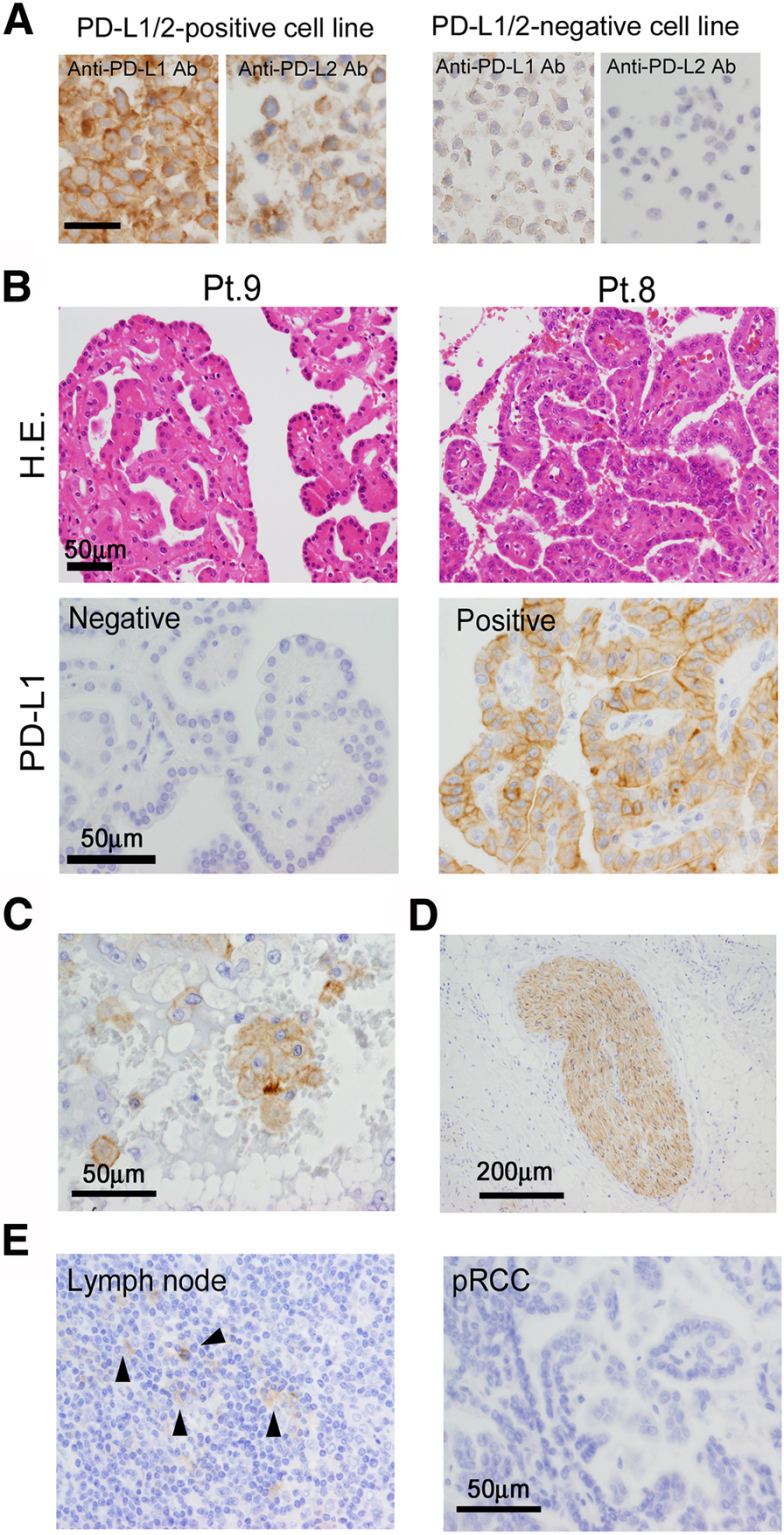
PD-L1/2 expression in pRCC **a** Pictures of immunostaining of PD-L1/2 in two cell lines (PD-L1/2-positive cell line; ATL-T, PD-L1/2-negative cell line; DAUDI) **b** Pictures of H.E. staining and immunostaining of PD-L1 in 2 cases of pRCC. Positive signals are detected most strongly in cancer cells **c** Positive signals for PD-L1 are also detected in macrophages **d** Positive signals for PD-L1 are also detected in neuronal fibers **e** PD-L2 is detected in dendritic cells in the lymph node, whereas no PD-L2 is observed in the cancer cells of any of the pRCC cases

At first, we confirmed the specificities of anti-PD-L1 and PD-L2 monoclonal antibodies used for immunostaining using cell block samples of two cell lines (Fig. 1a). In total, 102 resected specimens of pRCC were used for immunostaining of PD-L1/B7-H1; the characteristics of the patients are shown in Table 1. PD-L1-positive cancer cells were detected in 29 of the 102 cases, and the positive signals were detected on the cell surface membrane and cytoplasm of the cancer cells (Fig. 1b). Macrophages stained positive for PD-L1 in 4 cases (Fig. 1c), but no infiltrated lymphocytes were positive for PD-L1. Notably, neurons were strongly positive for PD-L1 in all cases (Fig. 1d). In contrast, no expression of PD-L2 was observed on any of the cancer cells or infiltrating macrophages and lymphocytes (Fig. 1e).

**Table 1:** Characteristic of pRCC patients

Gender/Male		80
Female		22
Age (Years)		
<70		60
≧70		42
Median (Years) 66		66
T classification		
T1/2		82
T3/4		20
Fuhrman grade		
G1/G2		72
G3/G4		30
Histological subtype		
Type 1		52
Type 2		50
Clinical course		
Reccurence		16
Cancer specific death 14		14

**Fig. 2:**
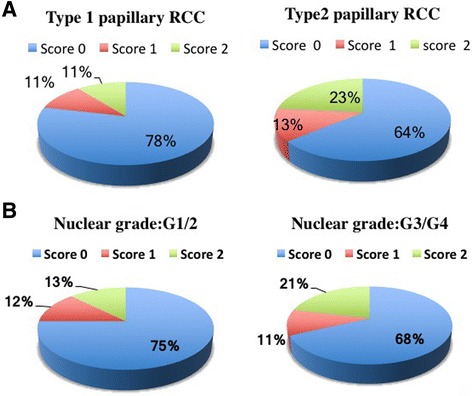
The frequencies of scores 0, 1, and 2 in pRCC. Cases were divided into two groups by histological subtype (**a**) or nuclear grade (**b**), and the frequencies of scores 0, 1, and 2 are shown

**Fig. 3:**
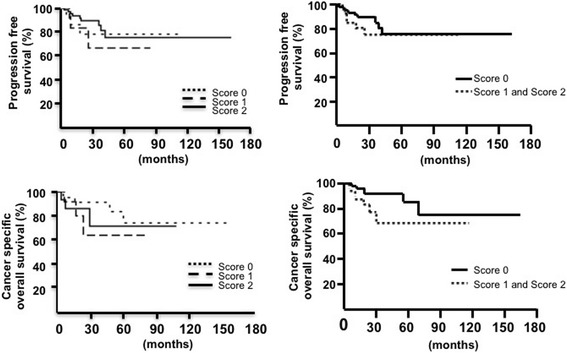
Kaplan-Meier analysis of cancer-specific overall survival and progression-free survival

Next, we classified the PD-L1 staining patterns into three scores according to the percentage of PD-L1-positive cancer cells: score 0, negative or less than 2%; score 1, 2 to 30%; and score 2, more than 30% (Fig. 2a, Additional file 1 table S1). Overall, the most frequent pattern was score 0 (71%), while score 1 and score 2 comprised 11 and 18%, respectively, of the cases. Since there are two histological subtypes of pRCC (types 1 and 2), the frequencies of scores 0 1 and 2 were compared between the subtypes. The frequency of score 1/2 (PD-L1-positive) was higher in type 2 (36%) than in type 1 (22%) pRCC; however, there was no significant difference in the percentages of score 0 cases (*p* value = 0.084 in Chi-square test, Fig. 2). Next, the frequencies of scores 0, 1 and 2 were compared between the nuclear grades. The frequency of score 1/2 (PD-L1-positive) was slightly higher in grade 3/4 (32%) than in grade 1/2 (25%); however, there was no significant difference between grade 1/2 and grade 3/4 (*p* value = 0.47 in Chi-square test, Fig. 2). The percentage of score 2 seemed to be higher in Type 2 (23%) or grade 3/4 (21%) than that in Type 1 (11%) or grade 1/2 (13%); however, however, there was no significant difference (*p* value = 0.42 or 0.36 in Chi-square test respectively, Fig. 2). Although score 0 cases seemed to have shorter progression-free survival and longer cancer specific overall survival, there was no significant correlation between PD-L1 scores and clinical course (Fig. 3).

## Discussion

Expression of PD-L1 is associated with a poor clinical course in colorectal cancer, lung cancer, ovarian cancer, and ccRCC [17–22]. In this study, we demonstrated that PD-L1 expression on cancer cells is not useful as a biomarker in pRCC. PD-L1 expression is positive in approximately 50% of ccRCC cases [13, 17, 18], which is much higher than in the pRCC cases of the present study. PD-L1 expression is known to be induced by infiltrating T cell-derived interferon γ. It is well known that ccRCC is an immunogenic cancer and many T-cell infiltrations are detected in ccRCC [23]. However, T-cell infiltration was lower in pRCC than in ccRCC in our preliminary observations (data not shown). PD-L1 expression and the density of infiltrating CD8-positive T cells have been shown to be well correlated in ccRCC [24]. The differences in the frequencies of PD-L1-positive cells between ccRCC and pRCC might be due to the immunogenicity of the cancer cells.

PD-L1 expression is also known to be detected in infiltrating leukocytes, including macrophages [25]; however, PD-L1 expression was observed only in macrophages in 4 of the 102 cases. PD-L1 expression in macrophages is induced by cancer-derived factors, and macrophage-derived interleukin 10 is involved in the induction of PD-L1 expression [26], suggesting that cell-cell interaction with cancer cells is necessary for PD-L1 expression in macrophages. The density of macrophages is closely associated with poor clinical prognosis in ccRCC [27], but no report has yet described the relationship between macrophages and clinical course in pRCC. The discrepancy in PD-L1 expression in macrophages between ccRCC and pRCC might be due to the induction of cell-cell interactions between cancer cells and macrophages.

Although PD-L2 expression in cancer cells has been reported in ovarian cancer [16], there are much fewer studies on PD-L2 than on PD-L1 in cancer tissues. This might be due to the fact that no monoclonal antibody suitable for use on paraffin sections had been commercially available. However, a new monoclonal antibody against PD-L2 has recently been made commercially available [28], and as a result, some research articles related to PD-L2 expression have just been published. One such article stated that PD-L2 expression was seen in 49% of pRCC cases; however, no pictures were published [29]. The staining density of PD-L2 expression seemed to be lower than that of PD-L1 expression (Fig. 1a), therefore, appropriate positive or negative controls are required and should be presented in research articles to indicate whether the immunostaining procedure was correctly performed.

## Conclusion

In the present study, PD-L1 expression was observed in 28% of the pRCC cases. The frequency of score 2 was high in type 2 or higher nuclear grade cases. Although PD-L1 expression appeared to be related to worse overall survival, there was no significant correlation between PD-L1 expression and clinical prognosis. Further studies are necessary to evaluate if PD-L1 expression might be useful for predicting the efficacy of anti-cancer immunotherapy using immuno-checkpoint inhibitors.

## Additional file

Additional file 1: Table S1. The data of PD-L1 and clinicopatholgical factors (DOCX 30 kb)

## Abbreviations

PD-1: Programmed death-1
RCC: Renal cell carcinoma
ccRCC: Clear cell RCC
pRCC: Papillary RCC
CTLA-4: Cytotoxic T lymphocyte antigen 4
PD-L1/2: Programmed death-1 ligand 1/2
