# Supplementary material for: PD-L1 expression in papillary renal cell carcinoma
Source: BMC Urol. 2017 Jan 13;17:8. doi: 10.1186/s12894-016-0195-x (PMC5237189; doi:10.1186/s12894-016-0195-x)
Supplement: Additional file 1: Table S1. — The data of PD-L1 and clinicopatholgical factors (DOCX 30 kb) [file 12894_2016_195_MOESM1_ESM.docx]

| Pt. No. | PD-L1 Score 0,1,2 | Age  1;≧66±10.5  2:＜66±10.5 | Gender | Nuclear grade | Histological subtype Type 1 or 2 | T clasification | Recurrence | Outcome |
| --- | --- | --- | --- | --- | --- | --- | --- | --- |
| 1 | 0 | 1 | M | G3 | 1 | pT1a | yes | dead |
| 3 | 0 | 1 | M | G2 | 1 | pT1b | yes | dead |
| 4 | 0 | 1 | F | G2 | 2 | pT3b | yes | dead |
| 5 | 0 | 1 | F | G2 | 1 | pT1a | no | alive |
| 6 | 0 | 2 | M | G2 | 1 | pT1a | no | alive |
| 7 | 1 | 2 | M | G3 | 2 | pT1b | no | alive |
| 8 | 2 | 1 | M | G2 | 1 | pT1a | no | alive |
| 9 | 0 | 1 | F | G2 | 1 | pT1a | no | alive |
| 10 | 1 | 1 | M | G1 | 1 | pT1a | no | dead |
| 11 | 0 | 1 | M | G2 | 1 | pT1b | no | alive |
| 12 | 0 | 1 | M | G2 | 2 | pT1a | no | alive |
| 13 | 1 | 1 | M | G2 | 1 | pT1b | no | alive |
| 14 | 0 | 1 | M | G4 | 2 | pT3a | no | alive |
| 15 | 0 | 1 | M | G2 | 2 | pT1b | no | alive |
| 16 | 1 | 1 | M | G2 | 1 | pT2b | no | alive |
| 17 | 2 | 1 | F | G2 | 1 | pT1a | no | alive |
| 18 | 0 | 1 | M | G1 | 1 | pT1a | no | dead |
| 19 | 0 | 1 | M | G3 | 1 | pT1a | no | alive |
| 20 | 0 | 1 | M | G2 | 1 | pT1a | no | alive |
| 21 | 0 | 1 | M | G2 | 1 | pT1a | yes | alive |
| 22 | 1 | 1 | M | G2 | 1 | pT1a | no | alive |
| 23 | 0 | 1 | M | G3 | 1 | pT1a | no | alive |
| 24 | 2 | 2 | M | G3 | 1 | pT1b | yes | dead |
| 25 | 2 | 1 | M | G2 | 2 | pT1b | no | alive |
| 26 | 0 | 1 | M | G4 | 1 | pT4 | yes | alive |
| 27 | 0 | 1 | F | G3 | 1 | pT1a | no | alive |
| 28 | 0 | 1 | M | G2 | 1 | pT1a | no | alive |
| 29 | 0 | 1 | F | G1 | 1 | pT1a | no | alive |
| 30 | 0 | 1 | M | G3 | 1 | pT1a | no | alive |
| 31 | 0 | 1 | M | G2 | 1 | pT1a | no | alive |
| 32 | 0 | 1 | M | G2 | 1 | pT1a | no | alive |
| 33 | 0 | 1 | M | G2 | 2 | pT1a | no | alive |
| 34 | 0 | 1 | M | G3 | 1 | pT1a | no | alive |
| 35 | 1 | 1 | F | G2 | 1 | pT1a | no | alive |
| 36 | 0 | 1 | M | G2 | 1 | pT1a | no | alive |
| 37 | 0 | 1 | F | G2 | 2 | pT1a | no | alive |
| 38 | 0 | 1 | M | G2 | 1 | pT1a | no | alive |
| 39 | 0 | 1 | F | G1 | 1 | pT1a | no | alive |
| 40 | 0 | 2 | F | G3 | 1 | pT1a | no | alive |
| 41 | 0 | 1 | F | G3 | 2 | pT1a | no | alive |
| 42 | 0 | 1 | M | G2 | 2 | pT1a | no | alive |
| 43 | 0 | 1 | M | G3 | 1 | pT1a | no | alive |
| 44 | 0 | 1 | F | G3 | 2 | pT1a | no | alive |
| 45 | 0 | 1 | F | G2 | 1 | pT1a | no | alive |
| 46 | 0 | 1 | M | G2 | 1 | pT1b | no | alive |
| 47 | 0 | 1 | M | G3 | 2 | pT3b | no | alive |
| 48 | 2 | 1 | M | G3 | 2 | pT1a | no | alive |
| 49 | 0 | 1 | M | G3 | 2 | pT3b | no | alive |
| 50 | 0 | 1 | M | G2 | 1 | pT1a | no | alive |
| 51 | 2 | 1 | M | G2 | 1 | pT1a | no | alive |
| 52 | 0 | 1 | M | G3 | 2 | pT1a | no | alive |
| 53 | 2 | 1 | F | G3 | 2 | pT1a | no | alive |
| 54 | 0 | 1 | M | G2 | 1 | pT1a | no | alive |
| 55 | 0 | 1 | M | G1 | 1 | pT1b | no | alive |
| 56 | 1 | 1 | F | G3 | 2 | pT3a | yes | dead |
| 57 | 0 | 1 | M | G3 | 2 | pT1a | no | alive |
| 58 | 0 | 1 | M | G3 | 2 | pT3a | yes | dead |
| 59 | 2 | 1 | M | G3 | 2 | pT1b | no | alive |
| 60 | 2 | 1 | F | G1 | 2 | pT1b | no | alive |
| 61 | 0 | 1 | F | G2 | 1 | pT1a | no | alive |
| 62 | 2 | 1 | M | G3 | 2 | pT3a | no | alive |
| 63 | 0 | 1 | M | G3 | 2 | pT1b | yes | dead |
| 64 | 2 | 1 | M | G3 | 2 | pT1a | no | alive |
| 65 | 2 | 2 | M | G3 | 2 | pT2 | no | alive |
| 66 | 0 | 1 | M | G3 | 2 | pT3a | yes | dead |
| 67 | 0 | 1 | M | G3 | 2 | pT1a | no | alive |
| 68 | 0 | 1 | M | G2 | 1 | pT2 | no | alive |
| 69 | 0 | 1 | M | G2 | 1 | pT1a | no | alive |
| 70 | 0 | 1 | M | G3 | 2 | pT1a | no | alive |
| 71 | 0 | 1 | M | G3 | 2 | pT3a | no | alive |
| 72 | 0 | 2 | M | G2 | 1 | pT1b | no | alive |
| 73 | 0 | 1 | M | G3 | 2 | pT3b | no | alive |
| 74 | 0 | 2 | M | G3 | 2 | pT1a | no | alive |
| 75 | 1 | 1 | M | G2 | 2 | pT3b | yes | dead |
| 76 | 0 | 1 | M | G2 | 1 | pT1b | no | alive |
| 77 | 0 | 2 | M | G2 | 2 | pT1a | no | alive |
| 78 | 0 | 1 | M | G4 | 1 | pT3a | yes | dead |
| 79 | 0 | 1 | M | G3 | 2 | pT3c | no | alive |
| 80 | 1 | 1 | M | G3 | 1 | pT3a | yes | dead |
| 81 | 0 | 2 | F | G2 | 1 | pT2 | no | alive |
| 82 | 0 | 2 | M | G3 | 2 | pT1b | no | alive |
| 83 | 0 | 2 | F | G2 | 1 | pT1a | no | alive |
| 84 | 2 | 1 | M | G3 | 2 | pT1a | no | alive |
| 85 | 2 | 2 | M | G2 | 2 | pT1b | no | alive |
| 86 | 2 | 1 | F | G3 | 2 | pT3a | yes | dead |
| 87 | 0 | 2 | M | G2 | 1 | pT1a | no | alive |
| 88 | 0 | 1 | M | G2 | 2 | pT1b | no | alive |
| 89 | 1 | 1 | M | G2 | 2 | pT1a | no | alive |
| 90 | 0 | 2 | M | G2 | 2 | pT1a | no | alive |
| 91 | 0 | 1 | M | G2 | 1 | pT1b | no | alive |
| 92 | 0 | 1 | M | G3 | 1 | pT3a | no | alive |
| 93 | 0 | 1 | M | G3 | 2 | pT1b | no | alive |
| 94 | 2 | 2 | M | G3 | 1 | pT3a | yes | dead |
| 95 | 0 | 1 | M | G3 | 2 | pT1a | no | alive |
| 96 | 0 | 2 | M | G2 | 1 | pT1a | no | alive |
| 97 | 0 | 2 | F | G3 | 1 | pT1b | no | alive |
| 98 | 0 | 1 | F | G2 | 2 | pT1a | no | alive |
| 99 | 1 | 1 | M | G3 | 2 | pT3a | no | alive |
| 100 | 0 | 1 | M | G2 | 1 | pT1a | no | alive |
| 101 | 0 | 1 | M | G2 | 1 | pT1a | no | alive |
| 102 | 1 | 2 | M | G4 | 2 | pT3a | yes | dead |
